# Ferroptosis in Osteoarthritis: Towards Novel Therapeutic Strategy

**DOI:** 10.1111/cpr.13779

**Published:** 2024-12-03

**Authors:** Yiming Zhang, Jing Li, Jiane Liu, Yan Gao, Kehan Li, Xinyu Zhao, Yufeng Liu, Daijie Wang, Xiao Hu, Zheng Wang

**Affiliations:** ^1^ Department of Genetics and Cell Biology, School of Basic Medicine Qingdao University Qingdao China; ^2^ Department of Reproductive Medicine The Affiliated Hospital of Qingdao University Qingdao China; ^3^ Department of Hematology Rizhao People's Hospital Rizhao China; ^4^ Department of Hematology The Affiliated Hospital of Qingdao University Qingdao China; ^5^ International Joint Laboratory of Medicinal Food R&D and Health Products Creation/Biological Engineering Technology Innovation Center of Shandong Province Heze Branch of Qilu University of Technology (Shandong Academy of Sciences) Heze China; ^6^ Key Laboratory of Basic and Translational Research on Immune‐Mediated Skin Diseases; Jiangsu Key Laboratory of Molecular Biology for Skin Diseases and STIs Institute of Dermatology, Chinese Academy of Medical Sciences and Peking Union Medical College Nanjing China

**Keywords:** ferroptosis, mechanism, osteoarthritis, therapeutic strategy

## Abstract

Osteoarthritis (OA) is a chronic, degenerative joint disease primarily characterised by damage to the articular cartilage, synovitis and persistent pain, and has become one of the most common diseases worldwide. In OA cartilage, various forms of cell death have been identified, including apoptosis, necroptosis and autophagic cell death. Ever‐growing observations indicate that ferroptosis, a newly‐discovered iron‐dependent form of regulated cell death, is detrimental to OA occurrence and progression. In this review, we first analyse the pathogenetic mechanisms of OA by which iron overload, inflammatory response and mechanical stress contribute to ferroptosis. We then discuss how ferroptosis exacerbates OA progression, focusing on its impact on chondrocyte viability, synoviocyte populations and extracellular matrix integrity. Finally, we highlight several potential therapeutic strategies targeting ferroptosis that could be explored for the treatment of OA.

## Graphical abstract

Osteoarthritis (OA) is a chronic degenerative joint disease mainly characterised by articular cartilage damage, synovitis and persistent pain, and has become one of the most common diseases worldwide. In OA cartilage, various forms of cell death have been identified, including apoptosis, necroptosis and autophagic cell death. Ever growing observations indicate that ferroptosis, a newly‐discovered iron‐dependent form of regulated cell death, plays a detrimental role on OA occurrence and progression. In this review, we analyse the pathogenetic mechanisms of OA by which iron overload, inflammatory response and mechanical overload lead to ferroptosis. We then explore the contribution of ferroptosis to OA progression from aspects of chondrocytes attenuation, synoviocytes reduction and extracellular matrix degradation. Several potential therapeutic substances are discussed and summarised. Finally, we will talk about the challenges and future perspectives in this field.

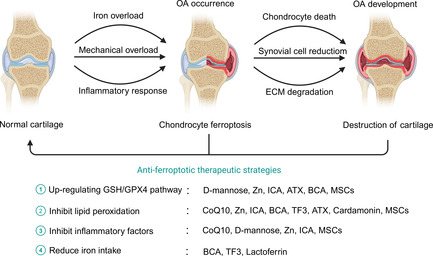

We believe that this review will improve the understanding of ferroptosis regulation in OA, and is crucial for comprehending normal physiology and the pathophysiology of OA, paving the way for powerful therapeutic strategies. As OA manifests through various mechanisms, particularly in inflammatory settings, this exploration holds promise for the development of novel diagnostic and therapeutic approaches in the field of OA.

## 1 Introduction

Osteoarthritis (OA) has become one of the most common diseases, affecting more than 500 million people worldwide [1]. This chronic degenerative disease compromises the entire synovial joint with key features of articular cartilage destruction, synovial inflammation, subchondral bone remodelling, osteophyte formation, joint space narrowing and tendons and ligaments instability [2, 3]. In severe cases, OA can lead to functional disability, severely diminishing the quality of life [4]. The pathogenetic mechanisms of OA is heterogeneous and intricate, and the link between OA risk factors and its development remains poorly understood. Multiple factors are considered to be involved in OA occurrence, including ageing, sex, obesity, heredity, hypertension, high‐intensity exercise, trauma and history of joint injury. These risk factors are proposed to lead to dysregulation of several key signalling pathways that maintain the cartilage homeostasis, leading to chondrocyte cell death, a typical hallmark of OA progression [5]. Apoptosis, necrosis and autophagy have been implicated in OA via different mechanisms reviewed elsewhere [6]. Of particular note, recently, a unique type of cell death with distinct morphological, biochemical, and genetic features has been identified in OA. Typically, this chondrocyte cell death is associated with aberrant mitochondrial ultrastructure, massive intracellular accumulation of iron‐dependent ROS, and, most notably, lipid peroxidation occurring in the plasma membrane accompanied by cellular membranes destruction [7], hinting at the potential involvement of ferroptosis in OA pathogenesis.

**FIGURE 1:**
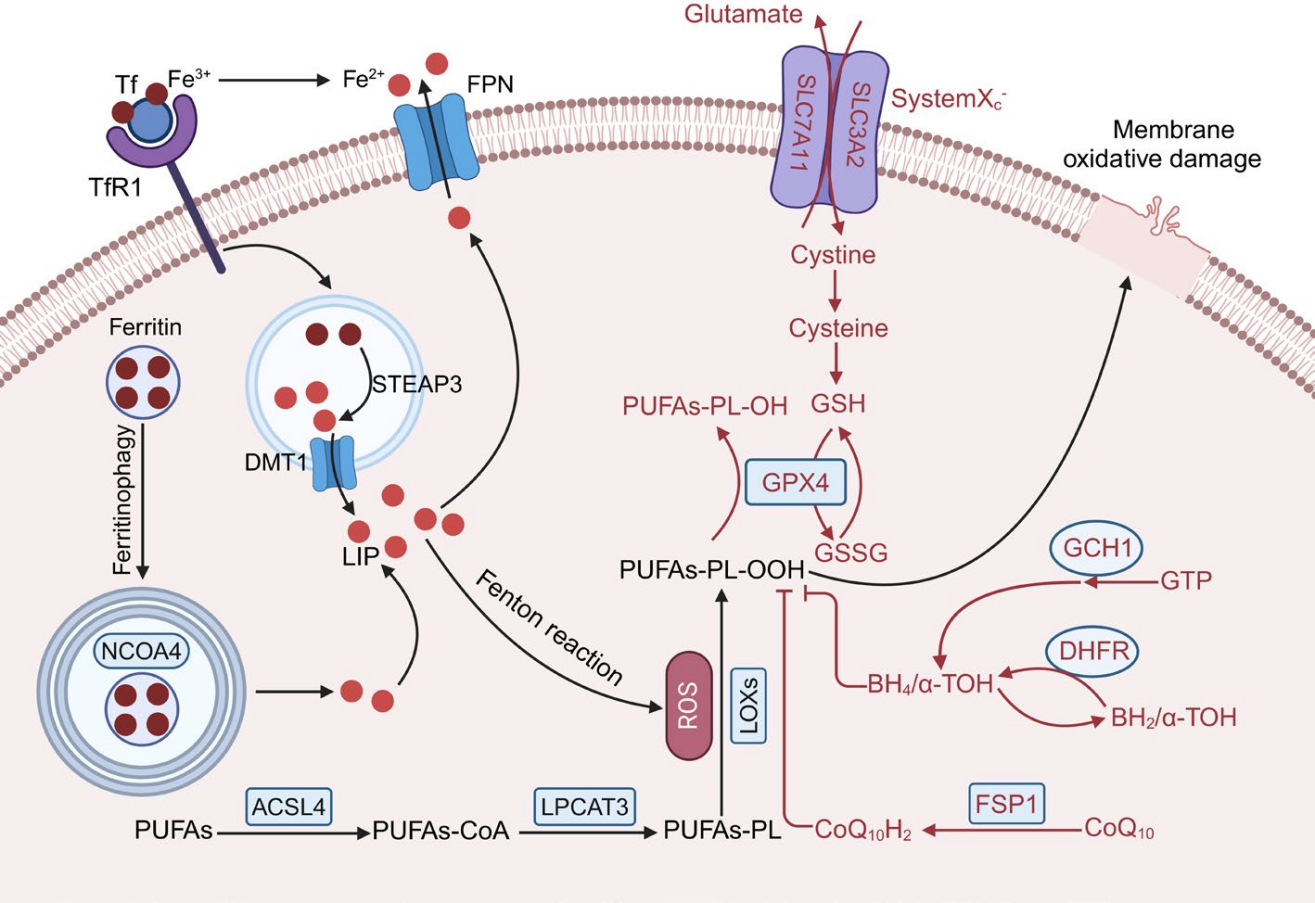
The Ferroptosis Signalling Pathway. Extracellular Fe^3+^ binds to transferrin (Tf) and crosses cells via transferrin receptor 1 (TfR1). Within endosomes, Fe^3+^ is released from Tf and subsequently reduced to Fe^2+^ by six‐transmembrane epithelial antigen of prostate (STEAP3). Fe^2+^ is then transported into cytoplasmic labile iron pool (LIP) via divalent metal transporter 1 (DMT1). The vital but toxic Fe^2+^, when excessive, is oxidised and stored as Fe^3+^ by ferritin. Nuclear receptor coactivator 4 (NCOA4) binds to ferritin and aids in its transport to the autophagosome for subsequent degradation, a process termed ferritinophagy. The export of intracellular Fe^2+^ into the extracellular space is mediated by ferroportin (FPN). Extensive production of reactive oxygen species (ROS) can be induced through the Fenton reaction, due to the redox ability of iron and stimulation of iron‐containing enzymes, such as lipoxygenases (LOXs). These abundant ROS cause damage to cell membranes, primarily through polyunsaturated fatty acids (PUFAs) containing carbon–carbon double bonds. PUFAs are esterified into PUFAs‐CoA under the action of long‐chain‐fatty‐acid—CoA ligase 4 (ACSL4), and then converted to PUFAs‐PL. PUFAs‐PL is oxidised to the lipid peroxide PUFAs‐PL‐OOH under the action of ROS and deposited. Multiple antioxidant defence systems were identified, including Xc‐/GSH/GPX4, FSP‐CoQ10‐NADPH and GCH1‐BH4‐DHFR. The xc‐system transfers equivalent cystine and glutamate into and out of cells, respectively, via SLC3A2 and SLC7A11 complex transporters. Cystine is then converted to cysteine and finally to GSH. Glutathione peroxidase 4 (GPX4), which converts lipid hydroperoxides (LOOH) into the non‐toxic lipid alcohols (LOH) by oxidising reduced glutathione (GSH) to oxidised glutathione (GSSG), leading to decreased PUFAs‐PL‐OOH to minimise cytotoxicity to the cells. Independent of GPX4, the FSP1‐CoQ10‐NADPH pathway defends against oxidation by taking advantage of ubiquinol (CoQ10H2), the reduced form of coenzyme Q10 (CoQ10), to sequester lipid peroxides as a lipophilic radical‐trapping antioxidant (RTA). The GCH1‐BH4‐DHFR pathway features the potent RTA tetrahydrobiopterin (BH4), with GTP cyclohydrolase 1 (GCH1) as the rate‐limiting enzyme for BH4 biosynthesis and dihydrofolate reductase (DHFR) as the enzyme required for BH4 regeneration.

Ferroptosis was first proposed by Dixon in 2012 as a novel form of regulated cell death specifically dependent on intracellular iron [8]. Iron, a crucial trace element, is involved in various physiological processes, including haemoglobin synthesis and oxygen transport. Under normal physiological conditions, cellular iron homeostasis is tightly regulated. Extracellular Fe^3+^ binds to transferrin (Tf) and crosses cells via transferrin receptor 1 (TfR1) [9]. Within endosomes, Fe^3+^ is released from Tf and subsequently reduced to Fe^2+^ by six‐transmembrane epithelial antigen of prostate (STEAP3) [10]. Fe^2+^ is then transported into the cytoplasmic labile iron pool (LIP) via divalent metal transporter 1 (DMT1) [11]. Since Fe^2+^ is vital but toxic, it is oxidised and stored in the form of Fe^3+^ by the 24 subunit iron storage protein ferritin when its concentration reaches a certain threshold [12]. Additionally, iron export can occur via ferroportin (FPN), which transports Fe^2+^ out of the cell. Under conditions of iron deficiency, nuclear receptor coactivator 4 (NCOA4) serves as a selective cargo receptor, binding to ferritin and aiding in its transportation to the autophagosome for subsequent degradation, a process termed ferritinophagy [13]. Such process results in the liberation of iron and restoration of Fe^2+^ concentration in the cytoplasmic LIRemarkably, disruptions in cellular iron homeostasis owing to increased iron absorption, loss of iron storage or reduced iron efflux, leads to accumulation of cytoplasmic iron, and consequently extensive production of reactive oxygen species (ROS) via Fenton reaction, a process catalysed by iron‐containing enzymes, such as lipoxygenases (LOXs) [14]. Massive ROS mainly takes the membranous carbon–carbon double bond‐containing polyunsaturated fatty acids (PUFAs) as targets, a process termed lipid peroxidation highly damaging to the plasma membrane. During this process, PUFAs are esterified into PUFAs‐CoA by ACSL4, and then the acyl groups are shed under the action of LPCAT3, producingPUFAs‐PL. The latter is accepted by ROS as a substrate to generate PUFAs‐PL‐OOH, a lipid peroxide that undermines the integrity of the plasma membrane. Apart from this pathway, lipid peroxides can be directly generated by LOXs‐mediated oxidation [15]. Ultimately, uncontrolled production of lipid peroxides in the membrane leads to ion mediated‐oxidative damage‐induced cell death, or ferroptosis (Figure 1).

To keep lipid peroxidation in check, cells have developed multiple antioxidant systems, among which Xc‐/GSH/GPX4, FSP1‐CoQ10‐NADPH and GCH1‐BH4‐DHFR are the major three pathways. The Xc‐/GSH/GPX4 is the primary pathway governing ferroptosis in mammals [16]. The xc‐system transfers cystine into the cell and transports equivalent amounts of glutamate out, via SLC3A2 and SLC7A11 transporters, respectively. Cystine is a precursor for the synthesis of glutathione (GSH) [17], which can be oxidised into glutathione disulphide (GSSG) by the selenoprotein glutathione peroxidase 4 (GPX4). During this enzymatic reaction, lipid hydroperoxides (LOOH) are reduced into the non‐toxic lipid alcohols (LOH), thereby lowering the risk of cytotoxicity from PUFAs‐PL‐OOH [18]. In parallel, the FSP1‐CoQ10‐NADPH pathway defends against oxidation by adopting ubiquinol (CoQ10H2), the reduced form of CoQ10, to sequester lipid peroxides as a lipophilic radical‐trapping antioxidant (RTA) [19]. Similarly, the GCH1‐BH4‐DHFR pathway utilises tetrahydrobiopterin (BH4) as another potent RTA, whose biosynthesis and regeneration are controlled by triphosphate cyclohydrolase 1 (GCH1) and dihydrofolate reductase (DHFR), respectively [20, 21] (Figure 1).

Ferroptosis has been well‐documented as a prominent feature of neurodegenerative disease, and intriguingly, strategies targeting ferroptosis have demonstrated significant beneficial effects [22, 23]. In recent years, ferroptotic iron accumulation and oxidative stress have been generally viewed as the universal pathological features of OA, underscoring the cruciall role of ferroptosis in this disease's progression. In this review, we summarise the underlying mechanisms of key factors in triggering ferroptosis that contribute to OA development. Finally, we provide a concise overview of ongoing endeavours aimed at targeting ferroptosis as a potential therapeutic vulnerability in OA.

## 2 Ferroptosis Induction in OA

Accumulating evidence emphasises the fact that ferroptosis serves as the nexus of multiple risk factors, including elevated dietary iron intake, ageing, hereditary disease, obesity and inflammatory condition. These factors are linked to mechanisms involving iron overload, mechanical stress and inflammatory responses leading to a risk of ferroptosis, and are presumed to underlie OA occurrence.

### 2.1 Iron Overload

**FIGURE 2:**
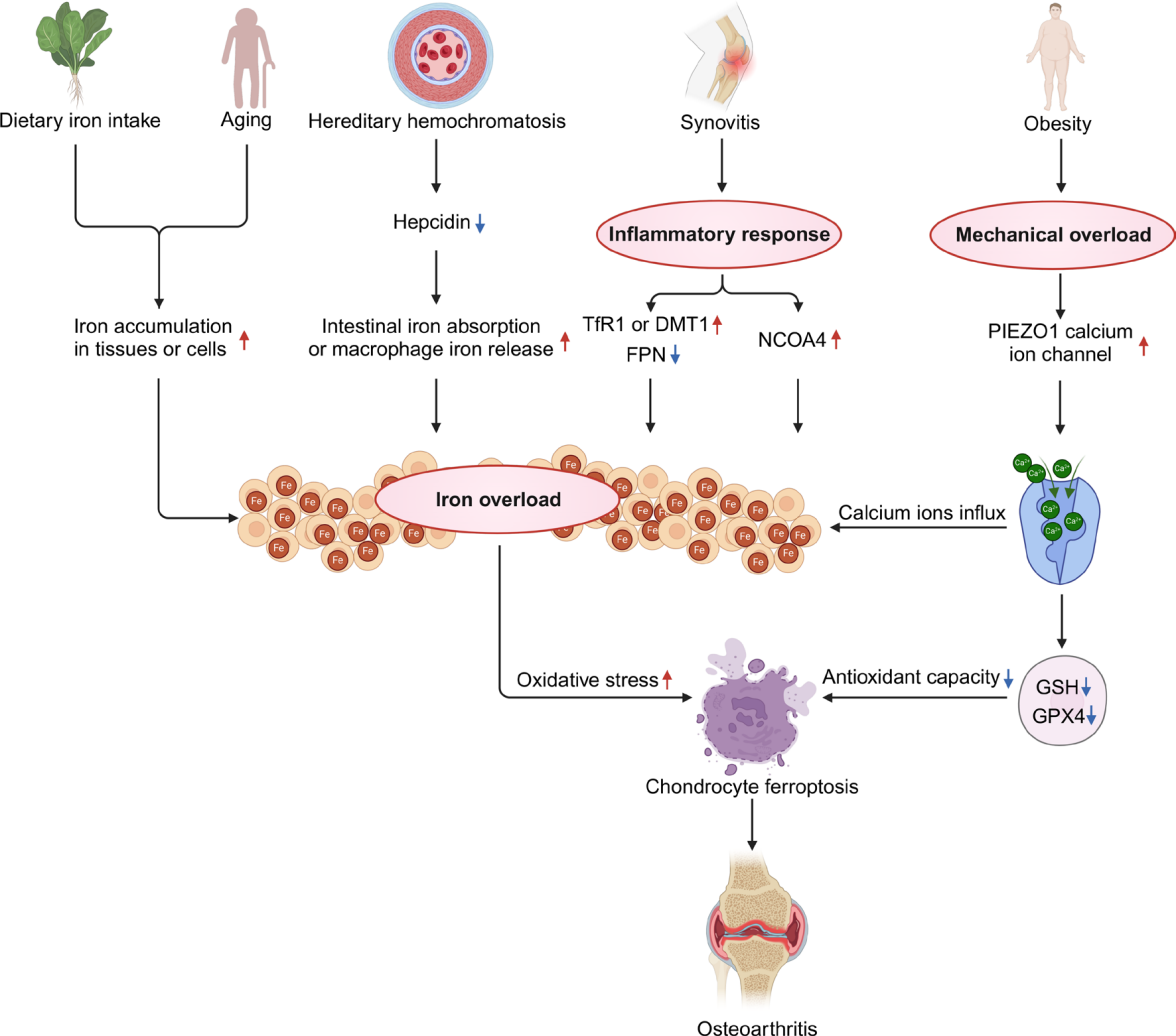
The induction of ferroptosis in OA. The ferroptosis in OA can be triggered through various mechanisms. The primary cause of chondrocyte ferroptosis in OA is iron overload, which may result from factors such as dietary iron intake, ageing, hereditary hemochromatosis (HH), synovitis, and obesity. In addition to inducing iron overload, obesity can also downregulate GSH and GPX4, further contributing to chondrocyte ferroptosis in OA.

An imbalance in intracellular iron homeostasis has been implicated in various diseases involving ferroptosis, such as liver disease, cardiovascular diseases, diabetes and neurological disorders [24, 25, 26, 27]. Notably, intercellular iron overload is a common feature among individuals with excessive dietary iron intake, ageing, or those diagnosed with hereditary hemochromatosis (HH) [28]. These groups are notably at an increased risk of developing OA (Figure 2). Remarkably, iron‐overloaded mice exhibit a greater degree of cartilage degeneration with higher Osteoarthritis Research Society International (OARSI) scores, and, importantly, treatment with deferoxamine (DFO), an iron chelator, reduces the severity of OA‐associated cartilage lesions in the knees of Dunkin‐Hartley guinea pig models, characterised by chondrocyte hypercellularity [29], highlighting iron overload as a major contributor to ferroptosis in cartilage degeneration in OA.

A large prospective longitudinal study with a 9‐year follow‐up period reported a strong correlation between OA progression and dietary iron intake in patients with OA [30]. Mechanistically, dietary supplementation or regular consumption of food rich in bioavailable iron results in large elevation in serum iron content and tissue iron storage due to the incapability of excreting excess iron, thereby triggering iron overload‐induced ferroptosis in chondrocytes [31] (Figure 2). As noted above, in Hartley guinea pig models fed with iron deficient diet, lower histologic OA scores and attenuated subchondral bone mineral density were detected accompanied by decreased iron concentrations in both serum and articular cartilage [32]. Immunohistochemistry assay revealed a marked decrease in the level of 4‐hydroxynonenal (4‐HNE, a main secondary product of lipid peroxidation and currently considered as a reliable bioactive marker of oxidative stress‐induced lipid peroxidation) [33] in both the menisci and articular cartilage of iron‐deficient group. These observations suggest that iron metabolism shapes iron‐dependent lipid peroxidation towards ferroptosis.

### 2.2 Mechanical Overload

Several meta‐analyses examining the relationship between body mass index (BMI) and the OA risk have emphasised obesity as a robust risk factor to OA, and remarkably, exercise interventions seem effective in alleviating OA symptoms by reducing pain and improving physical function as a result of reduction in body weight, BMI and visceral fat [34, 35]. Of note, intraoperative images of cartilage samples collected from both loading and unloading zones in patients with OA showed substantially greater damage in the loading zones, implying that aggravated wearing and tearing in articular cartilage is involved in OA development [36]. Strikingly, ferroptotic features were detected in chondrocytes specifically in the loading zones, exhibiting thickened mitochondrial membranes and mitochondria shrinkage, as visualised by transmission electron microscopy (Figure 2). Additionally, a markedly decreased expression level of *GPX4* was noted [37].

Following investigations have unveiled diverse mechanisms linking obesity to ferroptosis in OA. A microarray study showed increased levels of Piezo1, a mechanically‐activated ion channel mediating calcium influx, in the loading zones of articular cartilage tissues obtained from OA patients and also in primary chondrocytes stimulated with mechanical overload [37]. The elevated calcium influx impaired GSH production and subsequently diminished *GPX4* activity, setting up a reduced capacity of Xc‐/GSH/GPX4 antioxidant defence system. This axis highlights the involvement of Piezo1 channel in OA‐related ferroptosis. Additionally, another finding exposed a dramatically negative correlation between *SLC3A2* expression in chondrocytes and obesity grade [38]. As a subunit in the Xc‐system, *SLC3A2* reduction leads to restricted uptake of cysteine, and thereby constraining GSH synthesis [39]. Nevertheless, how *SLC3A2* is regulated by mechanical signals remains unclear, notwithstanding the potential overlap between the mechanisms mediated by Piezo1 and *SLC3A2* [40].

### 2.3 Inflammatory Response

It is well documented that inflammation is actively involved in the pathogenesis of OA. During the inflammatory response, excessive amounts of immune cells infiltrate into the synovium and subsequently release OA‐specific cytokines and chemokines. Among them, interleukin‐1β (IL‐1β) and tumour necrosis factor‐α (TNF‐α) are two major pro‐inflammatory cytokines [41, 42]. In patients with symptomatic knee osteoarthritis (KOA), IL‐1β and TNF‐α levels were found to be markedly elevated [43]. When primary chondrocytes derived from neonatal mice were exposed to IL‐1β, intracellular ROS level was heavily increased and lipid peroxidation was significantly enhanced [44]. Remarkably, these cells demonstrated an obvious ferroptotic process characterised by discontinuous and shrinking mitochondria. This phenotype is also observed in mouse chondrogenic cells with IL‐1β treatment, implying that chondrogenesis is also compromised. Importantly, treatment of Ferrostatin‐1 (Fer‐1), a common ferroptosis inhibitor, attenuates these changes [45], suggesting that ferroptosis undermines both tissue integrity and tissue repair in the presence of inflammatory cytokines. These findings confer on ferroptosis a promising target in OA inflammatory settings.

Mechanistic studies uncover the induction of iron influx factors, including TfR1 and DMT1, along with suppression of iron efflux mediator FPN in primary mouse chondrocytes (MCC) when treated with IL‐1β or TNF‐α. The dysregulation of these iron influx factors interrupted iron homeostasis, resulting in chondrocytic iron accumulation [46, 47] (Figure 2). Likewise, in response to lipopolysaccharide (LPS)‐induced synoviocyte inflammation, TfR1 was largely upregulated at the protein level, accompanied by increased iron content and malondialdehyde (MDA) levels. Of note, MDA is the major secondary product of lipid peroxidation and commonly viewed as an oxidative stress biomarker in clinics [48]. Consequently, increased chondrocyte death is identified in the ferroptotic form. IL‐1β can also induce the expression of *NCOA4*, which interacts with the ferritin complex to initiate autophagic degradation of ferritin proteins [47]. As a result, large amounts of iron is released to induce chondrocyte ferroptosis and an OA‐like phenotype. Knockdown of *NCOA4* by RNAi partially reversed IL‐1β‐induced chondrocyte ferroptosis, as evidenced by reduced level of ferrous ions, lipid‐ROS and MDA (Figure 2). Importantly, JUN has been suggested as a mediator of IL‐1β induced *NCOA4* expression by directly binding to *NCOA4* promoter. Indeed, in IL‐1β‐stimulated chondrocytes, absence of JUN results in a complete loss of *NCOA4* expression and regression of the OA phenotype [49]. Taken together, these studies establish a link between inflammation and ferroptosis‐driven OA progression, emphasising JNK‐JUN pathway as a potential target OA treatment [50].

## 3 Contribution of Ferroptosis to OA

Cartilage degeneration stands as the primary hallmark of OA, and loss of protective cartilage is viewed as the principle cause of this disease [5]. Central to cartilage degeneration are two significant pathophysiological processes: chondrocyte death and extracellular matrix (ECM) degradation. Ferroptosis in the context of developing OA has been shown to significantly worsen the disease by instigating chondrocyte death and ECM degradation, with substantial inflammation occurring concurrently.

### 3.1 Chondrocyte Death

Indeed, a spectrum of chondrocyte death has been identified in OA, including apoptosis, pyroptosis, necroptosis and ferroptosis. Among these, ferroptosis turns out to be a predominant contributor to OA development, particularly in the presence of ROS and inflammation. Studies modelling OA using the chondrogenic cell line ATDC5 or cartilage fragment‐derived primary MCC revealed that TBHP‐induced oxidative stress significantly reduced the viability of both ATDC5 cells and MCC. However, ferroptosis inhibitors such as Fer‐1 and DFO reversed these detrimental effects [51]. When using high concentrations of TBHP to mimic the late stage of OA, these inhibitors exhibited a superior protective effect to the RIP1‐targeting necroptosis inhibitor necrostatin‐1 (Nec‐1) [52]. These findings provide robust evidence supporting the role of ferroptosis in chondrocyte death within the context of ROS‐associated OA pathogenesis [51].

**FIGURE 3:**
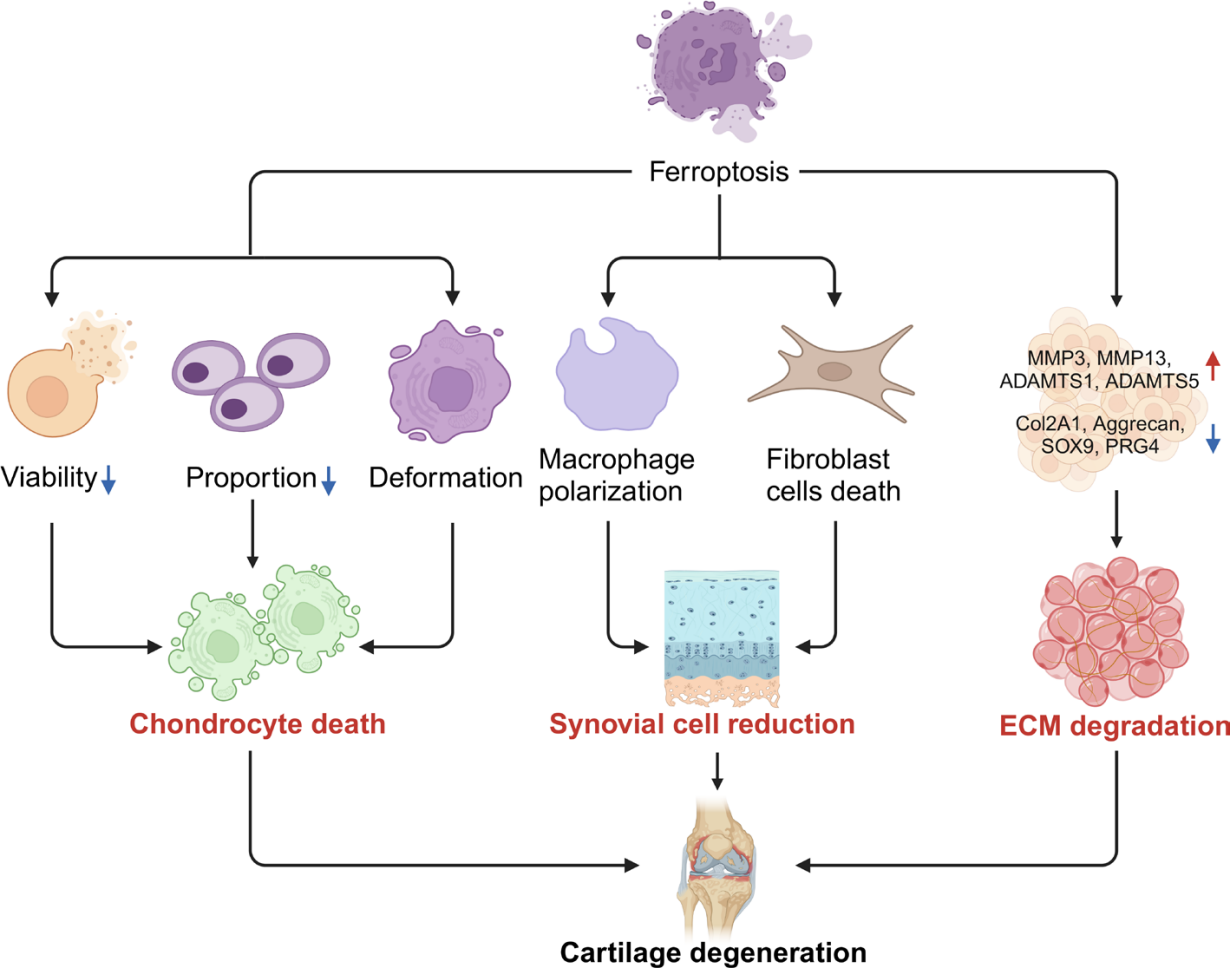
The contribution of ferroptosis to OA progression. In the occurrence of ferroptosis, substantial loss of chondrocytes can result from decreased cell viability, altered cell proportions and cellular deformation. Additionally, this process is associated with accelerated degradation of the extracellular matrix (ECM) due to the upregulation of matrix‐degrading enzymes and the downregulation of key components within the dense ECM structure in chondrocytes.

In another study, chondrocytes derived from post‐operative cartilage after total knee arthroplasty transformed from their ordinary triangular and closely arranged morphology into a branched spindle and sparsely arranged morphology in the presence of IL‐1β. These changes became increasingly pronounced after prolonged exposure, accompanied with dramatic reduction in chondrocyte proliferation and viability (Figure 3). Importantly, Fer‐1 reversed all the alterations induced by IL‐1β, indicating that ferroptosis underlies these cytotoxic activities [53]. Overall, these works underscore the relevance of ferroptosis in chondrocyte death during OA pathogenesis.

### 3.2 Synovial Inflammation

The synovium (also called synovial membrane) primarily consists of fibroblasts, macrophages, mast cells and adipocytes. Synovial inflammation is recognised as a critical contributor to OA pathophysiology, and ferroptosis has been shown to be a causal factor. The osteoarthritic synovial environment is characterised by elevated iron levels, with M2 macrophages more susceptible to ferroptosis than M1 macrophages (Figure 3). Notably, ferroptotic M2 macrophages released HMGB1, which interacted with TLR4 on M1 macrophages and drove M1 polarisation and inflammation activation [54]. This highlights the pivotal role of the HMGB1/TLR4/STAT3 axis in ferroptosis‐mediated inflammation in OA pathogenesis.

Another key cellular component of the synovium is fibroblasts, which are also prone to ferroptosis under inflammatory conditions (Figure 3). Treating fibroblast‐like synoviocytes (FLS) with Lipoxin A4 (LXA4), an endogenous anti‐inflammatory, pro‐resolving lipid mediator, inhibited ferroptosis in FLS and alleviated pain and pathological progression of KOA, possibly through activating the ESR2/LPAR3/Nuclear factor erythroid 2‐related factor 2 (Nrf2) axis [55]. Taken together, targeting ferroptosis‐related inflammatory pathways could serve as a promising strategy for OA treatment.

### 3.3 ECM Degradation

Cartilage ECM degradation is another hallmark of OA [56]. GPX4‐conditional knockout mice displayed increased osteophyte formation in the knee joints with exacerbated OA disease symptoms [56]. Molecular characterisation revealed a decrease in ECM protein Aggrecan and Collagen II, as well as an increase in matrix‐degrading enzymes MMP13 and ADAMTS5 [18] (Figure 3). This symbolises a shift towards matrix degradation under circumstances of ferroptosis in joints, posing a risk of losing essential components necessary for maintaining cartilage integrity.

In agreement, in the cartilage from anterior cruciate ligament transection (ACLT)‐induced OA mouse model, *MMP3*, *MMP13*, *ADAMTS1* and *ADAMTS5* were significantly upregulated, whereas *COL2A1*, *ACAN*, *SOX9* and *PRG4* were downregulated (Figure 3). Moreover, treating chondrocytes with IL‐1β in vitro reproduced these results. The fact that ferroptosis inhibitors Fer‐1 and DFO could reverse these changes both in vivo and in vitro strongly demonstrates that ferroptosis significantly contributes to ECM degradation and subsequent cartilage destruction [57].

## 4 Therapeutic Targeting Ferroptosis in OA

As ferroptosis gains recognition as a pivotal cell death mechanism in OA, targeting ferroptosis emerges as a promising strategy for its treatment. While Fer‐1 has demonstrated clear efficacy in inhibiting ferroptosis and alleviating OA symptoms, its clinical application is hindered by metabolic instability. Nonetheless, various RTAs known for preventing ferroptosis at its core mechanism have shown promising effects in the treatment of OA. Furthermore, the exploration of several cell‐based or nanoparticle‐based therapeutic strategies presents fascinating avenues for the treatment of OA (Table 1).

**TABLE 1:** Potential anti‐ferroptotic therapeutic strategies in OA.

Therapeutic strategy	Target	Experimental model	Mechanism of action	Reference
Co‐enzyme Q10	Lipid peroxides	LPS‐induced synoviocytes	Catalysed by FSP1 to ubiquinol to trap lipid peroxides	[19, 58]
D‐mannose	HIF‐2α	ACLT‐induced OA mouse model and IL‐1β‐induced primary mouse chondrocytes	Suppress HIF‐2α expression to promote expressions of GPX4 and SLC7A11 in Xc‐/GSH/GPX4 axis; scavenge ROS and inhibit lipid peroxidation	[63]
	IL‐1β	MIA‐induced OA mouse model	Suppress macrophage IL‐1β production to reduce inflammatory response	[66]
Zinc supplementation	IL‐1β and IL‐10	MIA‐induced chondrosarcoma SW1353 cells	Downregulate expressions of IL‐1β and IL‐10 to reduce inflammatory response	[70]
	PI3K	MIA‐induced chondrosarcoma SW1353 cells	Activate PI3K/Akt/Nrf2 pathway to promote expressions of antioxidant enzymes	[70]
	Nrf2	MIA‐induced chondrosarcoma SW1353 cells	Activate Nrf2 signalling pathway to enhance GSH synthesis; increase antioxidant capacity of Xc‐/GSH/GPX4 axis	[72]
Astaxanthin	iNOS and COX‐2	Human osteoarthritis chondrocytes	Improve the expression of inflammatory factors iNOS and COX‐2 to increase the expression of GPX4	[77]
Icariin	Nrf2	LPS‐induced human synoviocytes and IL‐1β‐induced human chondrocytes	Activate the Nrf2 signalling pathway to promote expressions of antioxidant enzymes; promote expressions of GPX4 and SLC7A11 in Xc‐/GSH/GPX4 axis; scavenge ROS and inhibit lipid peroxidation	[44, 72]
	IL‐1β	Human osteoarthritis fibroblast‐like synoviocytes	Downregulate IL‐1β expression to reduce inflammatory response	[41]
Biochanin	Nrf2 and GPX4	FAC‐induced mice primary chondrocytes	Activate Nrf2/System Xc‐/GPX4 signalling pathway; scavenge ROS and reduce oxidative stress	[86]
	TFR1 and FPN	FAC‐induced mice primary chondrocytes and iron dextrin‐induced iron overloaded OA mice model	Inhibit TfR1 expression and promote FPN expression to reduce LIP by	[86]
Theaflavin‐3,3‐digallate	Nrf2	Erastin‐induced human primary chondrocytes	Activate the Nrf2 signalling pathwayto promote expressions of GPX4, SLC7A11 and antioxidant enzyme HO‐1; scavenge ROS and inhibit lipid peroxidation	[89]
		Erastin‐induced human primary chondrocytes	Activate the Nrf2 signalling pathway to promote expression of FTH1 and increase iron storage; reduce intracellular iron accumulation to reduce iron‐induced ROS production and inhibit lipid peroxidation	[89]
Stigmasterol	SREBF2	IL‐1β‐induced mouse ATDC5 cells	Downregulate SREBF2; induces low expression of transferrin	[92]
	IL‐6 and TNF‐α	IL‐1β‐induced mouse ATDC5 cells	Downregulate pro‐inflammatory factors	[92]
Cardamonin	Nrf2	IL‐1β‐induced human chondrocytes	Activate antioxidant axis of Nrf2 to reduce the release of NO and PGE2	[97, 98]
Stem cell and exosome	HO‐1	Mouse pre‐osteoblast cell line, MC3T3‐E1 cells	Induce HO‐1 expression through the Nrf2/HO‐1 pathway	[111]
	METTL3	IL‐1β‐induced mouse OA chondrocytes	Disrupt the METTL3‐m6A‐ACSL4 axis and regulates the expression of ACSL4 by catalysing N6‐adenylate methylation of mRNA	[112]
	c‐MYC	MGC‐803 cells	Induce expression of c‐MYC and the expression of GSH	[113, 114]
Lactoferrin	Transferrin receptor	Osteocyte	Bind transferrin receptor and reducing cellular uptake of iron ions; inhibit inflammation	[115, 116]
PAA@Mn3O4‐NPs(PMO)	ROS	Acetaminophen and ischemia/reperfusion‐induced acute liver injury in mice	Eliminate ROS and inhibit iron mobilisation mediated by ferritin phagocytosis; activate mTOR pathway to suppress iron death	[117]
PDN@AG	Lipid and ROS	Intestinal ischemia‐reperfusion injury in mice	Reduce lipid peroxidation and ROS levels	[118]

### 4.1 RTAs Treatment in OA

#### 4.1.1 CoQ10

As a pivotal molecule within the FSP1‐CoQ10‐NADPH axis, which is one of the cellular antioxidant pathways involved in ferroptosis, CoQ10 undergoes catalysis by FSP1, resulting in its conversion into the reduced form, ubiquinol. This conversion enables the effective trapping of lipid peroxides as an RTA [19, 58]. Of such, intricately linked with combating ferroptosis, CoQ10 significantly impacts human health and disease that is reviewed elsewhere [59]. Notably, beyond mitigating cartilage damage attributable to ferroptosis, CoQ10 demonstrates a specific inhibitory effect on inflammation related to OA. Research conducted on rat articular chondrocytes and rat models illustrated CoQ10's capability to regulate NO levels and impede the activity of IL‐1β by inhibiting the MAPK pathway, both in vitro and in vivo [60, 61]. Despite minimal observed adverse effects, CoQ10 exhibits modest impact in the context of OA, probably lying in its ability to solely ameliorate ferroptosis through a singular pathway, lacking significant efficacy in alternative pathways [62].

#### 4.1.2 D‐Mannose

The role of hypoxia‐inducible factor 2α (HIF‐2α) in cartilage development and OA progression has been well‐established [63]. A recent focus on D‐mannose, a C‐2 epimer of glucose, has demonstrated its potential to suppress OA progression across various models [64]. Importantly, D‐mannose showed efficacy in downregulating the expression of HIF‐2α in chondrocytes [65]. The subsequent effect was the upregulation of *GPX4* and *SLC7A11*, critical suppressors of ferroptosis, leading to an increase in antioxidant capability and rendering chondrocytes more resistant to ferroptosis [66]. Moreover, the inhibition of HIF‐2α by D‐mannose was associated with a decrease in the expression of matrix‐degrading enzymes. This action contributed to the alleviation of cartilage damage by modulating the NF‐kB signalling pathway [66, 67]. Beyond its impact on HIF‐2α in chondrocytes, D‐mannose also inhibits succinate‐mediated HIF‐1α activation in macrophages. This dual effect resulted in the reduction of LPS‐induced IL‐1β expression by disrupting glucose metabolism and increasing intracellular mannose‐6‐phosphate levels [68]. However, it is essential to consider the potential side effects of D‐mannose, particularly its impact on glucose metabolism. Studies have suggested that its phosphate isomerase can impede glycolysis, leading to a reduction in ATP supply and potentially causing blindness in mice [69]. Despite these potential side effects, D‐mannose has demonstrated the ability to enhance chondrocyte autophagy and delay IL‐1β‐induced OA degeneration. This effect is mediated through GlcNAc‐6P, activating the AMPK pathway and ultimately inhibiting ferroptosis occurrence [70, 71].

#### 4.1.3 Zinc

Zinc, acting as a gatekeeper of the immune system, exerts an immunosuppressive effect in a monosodium iodoacetate (MIA)‐induced ferroptosis model by reducing pro‐inflammatory cytokines such as IL‐1β and IL‐10 [72]. The addition of zinc activated the PI3K/Akt/Nrf2 pathway, leading to the activation of downstream antioxidant enzymes, including GPx1, Zn/Cu‐SOD, Mn‐SOD and heme oxygenase‐1 (HO‐1). This activation helps regulate redox balance and subsequently decreases the production of ROS. Moreover, zinc induced the expression of glutamate‐cysteine ligase modifier subunit (GCLM) and catalytic subunit (GCLC), components of the GCL holoenzyme, which controls the rate‐limiting step in GSH biosynthesis. This enhanced the antioxidant capacity of the Xc‐/GSH/GPX4 axis [73]. Notably, Nrf2, activated by the PI3K/Akt pathway, directly targets *GPX4*, establishing a positive feedback loop to defend against ferroptosis [74]. However, an excess of zinc may advance OA progression by upregulating the metal‐regulatory transcription factor‐1 (MTF1), a zinc‐activated transcription factor responsible for inducing downstream matrix‐degrading enzymes in chondrocytes, including MMP3, MMP9, MMP12, MMP13 and ADAMTS5 [75, 76]. Additionally, the zinc importer ZIP8 was specifically upregulated in OA cartilage of humans and mice [77]. The complexity of these effects calls for caution in considering zinc supplementation, as both immunosuppressive and potentially detrimental effects on OA progression are associated with distinct zinc levels.

#### 4.1.4 Astaxanthin (ATX)

ATX, recognised as one of the most potent natural antioxidants, promotes bone health by exhibiting various beneficial effects. These effects include stimulating osteogenic markers, enhancing osteoblast differentiation, inhibiting osteoclast differentiation, increasing bone mineral density and reducing bone loss [78]. As expected, ATX demonstrated a significant anti‐ferroptotic effect in OA by facilitating the elimination of nitrogen compounds and sulphides, as well as mitigating free radical‐induced lipid peroxidation. Moreover, ATX modulated the expression of inflammatory factors such as iNOS and COX‐2, while increasing the expression of *GPX4* to further reduce oxidative stress. Additionally, ATX enhanced the Nrf‐2/HO‐1 signalling pathway, thus promoting mitochondrial autophagy, reversing mitochondrial membrane potential and improving the ECM to fight against ferroptosis [79, 80]. Besides, ATX harboured the ability to block Wnt signalling‐mediated ferroptosis through inhibiting the secreted Wnt agonist Rspo2 in chondrocytes, providing a protective mechanism against OA [81].

#### 4.1.5 Icariin (ICA)

ICA, a pharmacologically bioactive monomer extracted from *Herba epimedium*, demonstrates a range of beneficial effects, including anti‐inflammatory [82], antioxidative, anti‐ageing [83] and neuroprotective effects [84]. Notably, ICA exhibits a chondroprotective effect by targeting various molecules involved in the ferroptotic process [48]. Similar to zinc supplementation, ICA activated the Nrf2/ARE pathway, leading to the activation of downstream antioxidant enzymes such as SOD‐2, NQO‐1, and their downstream target genes *HO‐1*, *GPX4*, and *SLC7A11* in IL‐1β or LPS‐treated human chondrocytes [44, 74]. Additionally, ICA directly suppressed the expression of IL‐1β, thereby mitigating the progression of OA [41]. However, it is important to note that ICA may have associated adverse effects, including G2/M blockade‐induced decreases in bone resorption and BMP‐2/Smad4 upregulation which can result in the development of bone calcified nodules [85].

#### 4.1.6 Biochanin (BCA)

BCA, a bioactive natural compound isolated from *Huangqi*, has been shown to have multiple effects including anti‐inflammatory [86] and antioxidative [87] properties. BCA was initially associated with ferroptosis due to its neuroprotective effects against multiple neurological disorders [88]. Interestingly, recent findings indicated that it also displayed chondroprotective effects in ferric ammonium citrate (FAC)‐induced chondrocyte cell death model and iron overload‐induced OA mouse model [89]. Unlike other RTAs, BCA directly reduces LIP by inhibiting TfR1 expression and promoting FPN expression in a dose‐dependent manner, thereby restoring intracellular iron levels to prevent iron overload‐induced ferroptosis. Moreover, BCA activated Nrf2 to suppress the System Xc‐/GSH/GPX4 pathway, reducing ROS levels and lipid peroxidation, which might be accountable for the reduced cartilage degradation in the knee joints of BCA‐treated OA mice [89].

#### 4.1.7 Theaflavin‐3,3‐Digallate (TF3)

TF3 is a polyphenol compound extracted from black tea. It exhibits chondroprotective effects in both an erastin‐induced chondrocyte ferroptosis model and a rat OA model [90, 91], probably through activating the Nrf2/ARE pathway. In addition, it upregulated ferritin heavy chain 1 (FTH1), a component of ferritin that controls intracellular iron storage capacity, suggesting a potential role for TF3 in maintaining iron homeostasis and mitigating iron‐induced damage in chondrocytes [92]. However, whether TF3 exerts its protective effects by interfering inflammatory pathways remains to be clarified.

#### 4.1.8 Stigmasterol (STM)

STM has been identified as the active component of *Achyranthes bidentate* known as traditional Chinese medicine for its effectiveness in the treatment of KOA [93, 94]. Recent mechanistic studies revealed its role in attenuating IL‐1β‐induced upregulation of the sterol regulatory element‐binding factor‐2 (SREBF2) gene [95] in ATDC5 cells. Of note, SREBF2 single nucleotide polymorphism (SNP) was suggested as a risk factor for OA [96, 97]. Although the exact mechanism awaits further delineation, STM reduced SREBF2 abundance and hampered its involvement in the inflammatory response induced by IL‐1β [95], though SREBF2 has also been shown to paradoxically confer resistance to ferroptosis in circulating melanoma cells via directly activating the transcription of Tf [98].

#### 4.1.9 Cardamonin

Cardamonin, a chalcone compound derived from ginger plants, has demonstrated remarkable antioxidant and anti‐inflammatory properties [99]. Specifically, it suppressed oxidative stress via activation of the Nrf2 pathway [100, 101], and enhanced the functionality and structure of mitochondria via p53‐mediated upregulation of *GPX4* within chondrocytes [102]. Furthermore, cardamonin has been suggested to exhibit anti‐inflammatory properties by modulating NLRP3β and COX‐2 to inhibit IL‐1 [103]. However, studies also indicated that cardamonin was more easily absorbed by female rats [104], and it had a lower absorption rate, being excreted through faeces rather than urine [105].

#### 4.1.10 Mesenchymal Stem Cells‐Based Therapy in OA

Autologous chondrocyte implantation, introduced as the initial cell‐based surgical strategy for cartilage regeneration in 1994, faces limitations in addressing the complex degenerative changes seen in OA patients. The therapy demonstrated limited effectiveness due to the chondrocytes' restricted in vivo proliferative capacity and age‐related constraints, only being viable for patients under 40 years old [106, 107]. Additionally, monolayer‐cultured chondrocytes tended to dedifferentiate, leading to a loss of the ability to produce type II collagen [108]. In response to these challenges, MSCs emerge as an ideal cell type for OA treatment. MSCs, sourced from various tissues such as umbilical cord, cord blood, fat, bone marrow, synovium, synovial fluid and periosteum, have demonstrated promising attributes in clinical practise [109]. MSC derived from umbilical cord, adipose and bone marrow are commonly used for OA treatment, as they exhibited active immunosuppressive properties, capacity for chondrocyte differentiation, high proliferation potential and stability without undergoing dedifferentiation [110]. Pathologically, a plethora of preclinical and clinical studies have underscored the advantageous impact of MSCs on OA. These studies have consistently demonstrated several beneficial effects, encompassing mitochondrial repair, as well as anti‐inflammatory and immunomodulatory properties [111, 112, 113]. Notably, these observed effects imply a close association with ferroptosis.

The significant impact of MSCs on OA joint regeneration is primarily attributed to their paracrine stimulation of the local microenvironment. Various mechanisms have been proposed to elucidate how extracellular vesicles, particularly MSC‐derived exosomes, target ferroptosis: (i) **Induction of HO‐1 expression through the Nrf2/HO‐1 pathway:** histological analysis demonstrated that MSC‐derived exosomes inhibited ferroptosis and reduced inflammatory levels in a bilateral oophorectomy mouse model by inducing HO‐1 expression through Nrf2/HO‐1 pathway [114]. (ii) **Disruption of the METTL3‐m6A‐ACSL4 axis:** METTL3 regulated the N6‐methyladenosine (m6A) modification of ACSL4 mRNA, increasing ACSL4 mRNA stability and ACSL4 expression [115]. MSC‐derived exosomes, although the specific functional component remains unclear, reduced chondrocyte ferroptosis and prevented OA progression by disrupting the METTL3‐m6A‐ACSL4 axis [115]. (iii) **Regulation of GSH levels through the expression of c‐MYC:** MSC exosomes induced the expression of c‐MYC, a transcriptional regulator, which in turn activated the expression of GSH, thus regulating the levels of this important antioxidant [116, 117].

#### 4.1.11 Nanomedicine in OA

Recent advances in nanomaterials have shown potential in addressing OA by targeting ferroptosis. lactoferrin, for example, competitively binds to transferrin receptors, reducing cellular uptake of iron ions [118, 119]. This reduction in intracellular iron levels inhibits the inflammation triggered by ferroptosis, offering a promising therapeutic strategy for OA. Similarly, PAA@Mn₃O₄ nanoparticles (PMO), which accumulate in lysosomes, could scavenge ROS, inhibite ferritinophagy‐mediated iron release and maintained sustained activation of the mTOR pathway. While this approach was initially investigated in the context of acute liver injury, it also holds potential applications for OA treatment [120]. Additionally, other nanomaterials may be suitable for future OA therapies, although research specifically targeting ferroptosis inhibition in OA‐related conditions remains limited. For example, PDN@AGL showed protective effects in intestinal ischemia–reperfusion injury by reducing lipid peroxidation and ROS levels, thereby inhibiting ferroptosis [121].

## 5 Conclusion and Future Perspectives

The fields of ferroptosis and OA have recently intersected, unveiling a novel therapeutic strategy for OA. This convergence sheds light on the role of ferroptosis in the onset and progression of OA, emphasising the importance of understanding these underlying mechanisms at different stages of OA. Such knowledge is crucial for comprehending both normal physiology and the pathophysiology of OA. As OA manifests through various mechanisms, particularly in inflammatory contexts, this exploration offers promise for the development of novel diagnostic tools and therapeutic approaches (Table 1).

Ferroptosis exhibits intricate connections with various other cell death types through crosstalk mechanisms. This relationship is evident in the induction of ferroptosis, triggered by the breakdown of the mitochondrial electron transport chain during apoptosis, leading to the generation of ROS. In inflammatory conditions, such as those observed in diseases like SARS‐CoV‐2, HLH and sepsis induced by a combination of TNF‐α and IFN‐γ, a mixed form of cell death known as “PANoptosis” emerges [122, 123]. PANoptosis involves simultaneous activation of pyroptosis, apoptosis and necroptosis [124]. Additionally, these inflammatory cytokines may contribute to ferroptosis by downregulating key components of the anti‐ferroptosis system, specifically the xc‐GSH‐GPX4 axis. Indeed, OA complicates the landscape, revealing the presence of multiple cell death types, including apoptosis, necroptosis, autophagy, pyroptosis, necrosis, and, recently, ferroptosis [125]. Interventions targeting these pathways exhibit varying effects on alleviating OA symptoms. However, given the intricate and multifaceted nature of OA aetiology, the extent to which ferroptosis predominates as a mechanism remains uncertain.

In addition to the primary triggers of ferroptosis in OA (iron overload, mechanical overload, and inflammatory responses) discussed above, several other factors may further enhance ferroptosis in this context: (i) **Elevated levels of ROS**: These arise from mechanical stress, inflammatory responses and mitochondrial dysfunction, contribute significantly to ferroptosis by predisposing chondrocytes to oxidative damage, as these cells are particularly vulnerable to such stressors [126, 127, 128]. (ii) **Dysregulation of key antioxidant defence systems**: This includes the Xc‐/GSH/GPX4, FSP1‐CoQ10‐NADPH and GCH1‐BH4‐DHFR pathways, impairs the cell's ability to neutralise lipid peroxides, thereby increasing susceptibility to ferroptosis [129, 130, 131]. (iii) **Mitochondrial dysfunction**: This exacerbates ferroptosis by generating excessive ROS and disrupting cellular energy metabolism, leading to impaired antioxidant responses and further intensification of oxidative damage [132]. (iv) **Abnormal lipid metabolism**: Characterised by increased synthesis of PUFAs and their integration into membrane phospholipids, this enhances vulnerability to lipid peroxidation [133]. Furthermore, enzymes such as ACSL4 and LPCAT3 [134], which are upregulated in ferroptotic conditions, exacerbate lipid peroxidation and contribute to cell death.

Currently, first‐line clinical treatments for OA consist of oral medications and intra‐articular injections. Oral medications primarily include non‐steroidal anti‐inflammatory drugs (NSAIDs) such as ibuprofen, aspirin and celecoxib. In terms of ferroptosis, ibuprofen has been shown to downregulate the Nrf2 signalling pathway, inducing ferroptosis in glioblastoma cells [135], and aspirin has been found to trigger ferroptosis in models of diabetes and kidney disease [136]. In clinical practise, intra‐articular injections such as sodium hyaluronate and glucocorticoids are commonly used. Notably, hyaluronic acid‐based nanocomposites have demonstrated the ability to induce ferroptosis in various tumour models [137]. However, whether these medications could induce ferroptosis in OA requires further investigation, and the complexity of these ferroptosis‐inducing effects should incite caution. Interestingly, celecoxib has been shown to alleviate ulcerative colitis in mice by inhibiting ferroptosis, suggesting its potential to modulate this process in OA as well [138]. Additionally, cartilage‐protective agents such as glucosamine sulphate and chondroitin sulphate are frequently used in early‐stage OA. Chondroitin sulphate has been reported to upregulate Nrf2, alleviate joint pain, and potentially reduce ferroptosis [139]. To this end, celecoxib and chondroitin sulphate may serve as promising candidates for OA treatment, not only for their established therapeutic effects but also for their potential to target ferroptosis.

While several anti‐ferroptotic agents are currently under clinical investigation for neurodegenerative diseases that is reviewed elsewhere [140], the potential efficacy of ferroptosis modulation in OA treatment is underscored by the complex interplay between ferroptosis and OA pathology. However, the prospect of utilising these modulators raises concerns regarding potential side effects, particularly given the limited understanding of ferroptosis's broader physiological functions, particularly in the context of the treatment of chronic conditions. For instance, D‐mannose supplementation can influence glycolysis, ATP supply and glucose metabolism or excessive Zn supplementation leading to copper deficiency, anaemia, leukopenia or prostate cancer. Moreover, the use of iron‐removing agents, such as DFO, deferiprone (DFP) and deferasirox (DFS), requires careful consideration [141]. While DFO exhibits potential in inhibiting ferroptosis in chondrocytes, its side effects, including allergic reactions and sensory impairments, warrant careful consideration [142]. DFP and DFS, primarily studied in tumours and aplastic anaemia, present challenges due to toxicities such as joint pain, agranulocytosis and acute renal failure, which limit their broad clinical application [143, 144]. Lastly, although CoQ10 did not demonstrate significant improvement in Parkinson's disease in a Phase 2 double‐blind, placebo‐controlled clinical trial [145], its therapeutic efficacy in the context of OA requires further investigation.

A comprehensive understanding of the dynamic control of ferroptosis in the OA setting is imperative. Most recent research has focused on elucidating the involvement of novel factors in ferroptosis in OA, including transcription factors such as Forkhead box O3 (FOXO3), specific protein 1 (Sp1), the capsaicin receptor Transient receptor potential vanilloid 1 (TRPV1), the membrane protein Semaphorin 5A (SEMA5A) and the RNA‐binding protein Staphylococcal Nuclease And Tudor Domain Containing 1 (SND1) [146, 147, 148, 149, 150]. These entities exert their influence through diverse mechanisms to modulate ferroptosis in the context of OA. Notably, microRNA‐1 has been identified as a key regulator that maintains the expression of *GPX4* and *SLC7A11* by targeting CX43 to inhibit the occurrence of ferroptosis, thereby imparting a protective effect on OA [151]. Understanding such intricate molecular interactions provides a basis for designing novel therapeutic strategies that target these molecules, potentially paving the way for more effective treatments for OA.

While stem cell therapy shows significant potential for cartilage regeneration in OA, its widespread clinical application encounters challenges, primarily due to issues such as tissue rejection and low survival rates in affected regions [152, 153]. In recent years, the utilisation of patient‐derived induced pluripotent stem cells (iPSCs) for generating differentiated MSCs has emerged as a promising approach to OA treatment, circumventing immune response concerns [154, 155]. Furthermore, recent research highlights the importance of superior bone tissue engineering materials for achieving effective in situ colonisation of cells, serving as a critical consideration in their development and application [156]. These scaffold materials can be categorised into natural, synthetic, composite, cartilage matrix and biological scaffolds. An ideal scaffold material, characterised by attributes such as tissue compatibility, biomolecule permeability and low immunogenicity, not only prolongs the local survival time of MSCs but also facilitates their differentiation into chondrocytes [156]. Incorporating materials that inhibit ferroptosis, such as chondroitin sulphate, which upregulate Nrf2, could further augment therapeutic efficacy. This tissue engineering approach presents an intriguing opportunity to integrate potential compounds (including anti‐ferroptotic agents as outlined in Table 1), further enhancing the anti‐ferroptotic effect, immune modulation or promoting MSC self‐renewal in vivo [157]. Consequently, this comprehensive strategy holds promise as a potential avenue for exploring treatments for OA caused by ferroptosis.

## Author Contributions

X.H. and Z.W. designed the manuscript. Y.Z and J.L. drafted the manuscript. J.L, Y.G. and K.L prepared the figures. X.Z and Y.L. created the table; D.W., X.H. and Z.W revised the manuscript. All authors contributed to the article and approved the final version for submission.

## Conflicts of Interest

The authors declare no conflicts of interest.

## Data Availability

Data sharing is not applicable because no new original data were generated for this review article.
